# Value of Oral Health Assessments for Older People with Memory Complaints Visiting a Memory Clinic for a Comprehensive Geriatric Assessment: A Cross-Sectional Study

**DOI:** 10.3390/ijerph23020212

**Published:** 2026-02-09

**Authors:** Sanne M. Pruntel, Lauren A. Leusenkamp, Arjan Vissink, Barbara C. van Munster, Anita Visser

**Affiliations:** 1 Department of Oral and Maxillofacial Surgery, University Medical Center Groningen and University of Groningen, Hanzeplein 1, 9713 GZ Groningen, The Netherlands; a.vissink@umcg.nl; 2 Section Gerodontology, Department of Dentistry and Orthodontics, Center for Dentistry and Oral Hygiene, University Medical Center Groningen and University of Groningen, 9713 AV Groningen, The Netherlands; l.a.leusenkamp@umcg.nl (L.A.L.); a.visser@umcg.nl (A.V.); 3 Department of Internal Medicine, University Medical Center Groningen and University of Groningen, 9713 GZ Groningen, The Netherlands; b.c.van.munster@umcg.nl

**Keywords:** oral health, cognitive complaints, memory clinic, comprehensive geriatric assessment, dementia, Alzheimer’s disease, older people

## Abstract

**Highlights:**

**Public health relevance—How does this work relate to a public health issue?**

- Cognitive decline and memory complaints are highly prevalent in ageing populations. Comprehensive geriatric assessments used in memory clinics often omit oral health, despite its known association with general health and quality of life.
- Oral health problems such as tooth decay, gum disease and denture-related issues are widespread and largely preventable, making them a significant yet under-recognized public health concern in older adults.

**Public health significance—Why is this work of significance to public health?**

- This study demonstrates that oral health problems are present in all patients with memory complaints, regardless of a dementia diagnosis. This indicates a substantial unmet need in current geriatric care pathways.
- By identifying oral health issues early within existing clinical assessments, there is potential to reduce avoidable complications, healthcare costs and further decline in overall health.

**Public health implications—What are the key implications or messages for practitioners, policy makers and/or researchers in public health?**

- Integrating structured oral health assessments into comprehensive geriatric assessments could improve the early detection of oral health issues and facilitate timely referrals, supporting a more preventive approach to care for older adults.
- The findings highlight the need for interdisciplinary collaboration and future research to develop standardized oral health screening protocols within geriatric and memory care settings.

**Abstract:**

The risk of memory complaints and cognitive decline increases with age, leading to many older adults being referred to memory clinics for cognitive screening including a comprehensive geriatric assessment (CGA). Although CGA covers medical, cognitive and social domains, it commonly omits an assessment of oral health, despite evidence linking poor oral health to cognitive impairment and reduced quality of life. This study assessed the value of incorporating an oral health assessment into the CGA and explored differences in oral health between patients with and without dementia. Patients with memory complaints attending a memory clinic between April 2022 and May 2025 were asked to undergo an oral health assessment and complete an oral health questionnaire alongside the CGA. Patients unable to cooperate or incapacitated to consent were excluded. In total, 144 participants were included. The mean age was 73.7 years; 59.7% were male. Most participants had natural dentition (75%), and most had carious lesions (93.5%) and periodontal pockets (97.3%). Among denture wearers, denture-related problems were common (61.7%). No differences in oral health were observed between patients with and without dementia. All participants had at least one oral health problem, regardless of whether or not they had dementia. Integrating structured oral health assessments into the CGA therefor has value as it offers opportunities for early detection, intervention, and prevention for further decline in overall and oral health.

## 1. Introduction

The risk of developing memory complaints and/or cognitive problems increases with age [1]. Memory complaints and/or cognitive problems refer to self- or informant-reported difficulties in memory or other cognitive areas, such as attention, executive functioning or language. These problems may interfere with daily life and can subsequently lead to a referral to a memory clinic. Therefore, people with memory complaints are often referred to a memory clinic to assess their cognitive status and whether they need help [2]. In a memory clinic, the general health and cognitive status of patients with memory complaints are assessed through a variety/battery of medical and neuropsychological tests, a hetero-anamnesis, and a radiologic investigation [2]. These assessments together aim to assess the severity and possible causes of the patient’s memory complaints and/or cognitive impairment as well as their functional and social needs [3]. This assessment is called a comprehensive geriatric assessment (CGA) [4].

Despite the fact that the CGA is considered a comprehensive assessment in the general health domain, including psychosocial domains, an assessment of oral health is omitted notwithstanding the growing evidence that oral health is linked to general health, mental health, social wellbeing and quality of life [5,6]. The importance of timely detection and management of poor oral health in older adults is highlighted by its association with malnutrition, frailty, systemic infections and reduced social participation [5,6]. It is well-known from the literature that older people with dementia, amongst other patients with Alzheimer’s Disease (AD), often have poor oral health: maintaining oral self-care and going to the dentist becomes difficult for them [7,8,9,10,11]. These oral health problems, such as periodontal disease, dental caries, and broken or mobile teeth, can cause infections, chewing problems and oral pain, which result in a higher risk of health problems and poorer (social) wellbeing [8,12,13].

Oral health problems are not confined to patients with established dementia. Older adults experiencing subjective or mild cognitive decline may already have signs of a deteriorating oral health and reduced dental attendance, indicating the need for earlier screening and intervention [12,13]. The current literature suggests that oral screening in geriatric care settings is inconsistent and is often only carried out in patients with advanced cognitive impairment or those in institutional care [7,8,10]. There is no information on the oral health status of older adults who attend memory clinics [9,10]. Integrating an oral health assessment into the CGA potentially has clear advantages, including the early identification of preventable oral and general health issues, the timely referral of patients to dental care and the opportunity to implement personalized preventive strategies for both dentate and edentulous patients, regardless of a dementia diagnosis [5,7,9].

Therefore, it would be a missed opportunity when oral health is not included in the CGA, particularly in patients with memory complaints visiting a memory clinic. Detecting oral health problems at an early stage of dementia is an opportunity to slow down or even prevent further decline in oral health and subsequently overall health including psychosocial function. However, it is unknown what the prevalence and the exact nature of oral health problems are in older people visiting a memory clinic. Therefore, we conducted a study whereby an oral health assessment was added to the CGA for patients referred to a memory clinic. The objective of this cross-sectional study was to assess the value of an oral health assessment during the comprehensive geriatric assessment of patients with memory complaints during their first visit to a memory clinic. It was also assessed whether patients diagnosed with dementia have more oral health issues than patients with memory complaints not diagnosed with dementia.

## 2. Materials and Methods

### 2.1. Study Design

A cross-sectional study was conducted to assess oral health in patients visiting the memory clinic for the first time because of memory complaints at the University Medical Center Groningen (UMCG) between April 2022 and May 2025. This tertiary referral university medical centre is located in the northern part of the Netherlands and serves both urban and rural populations in a wider region around the medical centre. All participants provided written informed consent for the study including the use of data from their medical records for additional analysis. This study was approved by the Institutional Review Board of the University Medical Centre Groningen (Netherlands research registration number 202000809). Informed consent was obtained from all patients.

### 2.2. Patient Population

All consecutive patients with memory complaints who were referred to the memory clinic of the UMCG and who were seen by a geriatrician for a diagnostic work up (CGA) were asked to join the study. Using a non-probability consecutive sampling approach, all consecutive patients attending the clinic during the study period were invited to participate. All patients were eligible to participate regardless of their sex, age or diagnosis. People who were unable to cooperate with a dental assessment due to their general condition (e.g., in case of severe cognitive problems or health issues) or people who were incapacitated were excluded. It was important that the participants were willing and able to follow instructions and to undergo an oral examination (see oral health assessment further on). During their visit to the memory clinic, all the participants’ demographic characteristics (age, gender, polypharmacy, smoking, alcohol use and comorbidities) were assessed.

### 2.3. Comprehensive Geriatric Assessment (CGA)

All patients who visit the geriatric outpatient clinic of the memory clinic routinely receive a CGA. This CGA of the UMCG includes an assessment of somatic conditions, medication, intoxications (smoking, drinking, drugs), a physical examination, and a routine laboratory blood testing. In addition, mental and functional status are tested. Included in this CGA is the Mini Mental State Examination (MMSE) or Montreal Cognitive Assessment (MOCA), assessment tools to screen patients for cognitive impairments [14]. The total score on the test ranges from 0 to 30 points, denoting an interval measurement level [14]. A score of 24 or lower is indicative of a cognitive disorder [14]. Finally, radiological examination with magnetic resonance imaging (MRI) or computed tomography (CT) of their brain is performed on indication of the geriatric specialist who performed the CGA. All tests that are performed within this CGA are specified per domain (somatic, mental, social and functional) in Table 1.

The results of all the tests together enable the geriatric team to diagnose the severity and cause for the cognitive problems patients are suffering from. After obtaining all the results of the various tests, patients are diagnosed by the geriatric team for diagnoses such as dementia, an undefined psychiatric disorder, a multifactorial condition (e.g., stress, anxiety, mood disturbances, low education, alcohol use, or non-congenital brain injury), mild cognitive impairment (MCI), cognitive impairment related to benzodiazepines, depression, anxiety disorder, or no clear etiology for the memory complaints. In some cases, further tests are needed. When possible, dementia is also subclassified as AD, frontotemporal dementia (FTD), Lewy body disease (LBD), vascular dementia (VD), primary progressive aphasia (PPA), posterior cortical atrophy (PCA), and corticobasal degeneration (CD). For the purpose of this study, two groups are considered: people with and without dementia.

### 2.4. Application of Questionnaires and Oral Health Assessment

In addition to the CGA, oral health was assessed in all patients who were willing to participate in this study, further mentioned as participants. After inclusion, all participants first received a questionnaire by mail or email to assess their oral situation (oral status; dentate, partial dentate, edentulous, wearing (implant retained) protheses), oral self-care (oral cleaning habits such as brushing, flossing, etc.) (Appendix A.1) and the Oral Health Related Quality of Life (Oral Health Impact Profile (OHIP-14) [18]. The questionnaire about the oral situation is not validated. The OHIP-14 questionnaire consists of 14 questions divided into seven categories: functional limitations, physical pain, psychological discomfort, psychological disability, physical disability, social disability and handicap. Each question is answered on a scale of 0 to 4, with 0 indicating that the problem never occurs and 4 indicating that the problem occurs very often. The OHIP-14 total score can reach a maximum of 56, with higher scores corresponding to lower oral health-related quality of life (Appendix A.2). This questionnaire has been previously validated. We checked all given information on self-reports with the informal carers.

**Figure 1:**
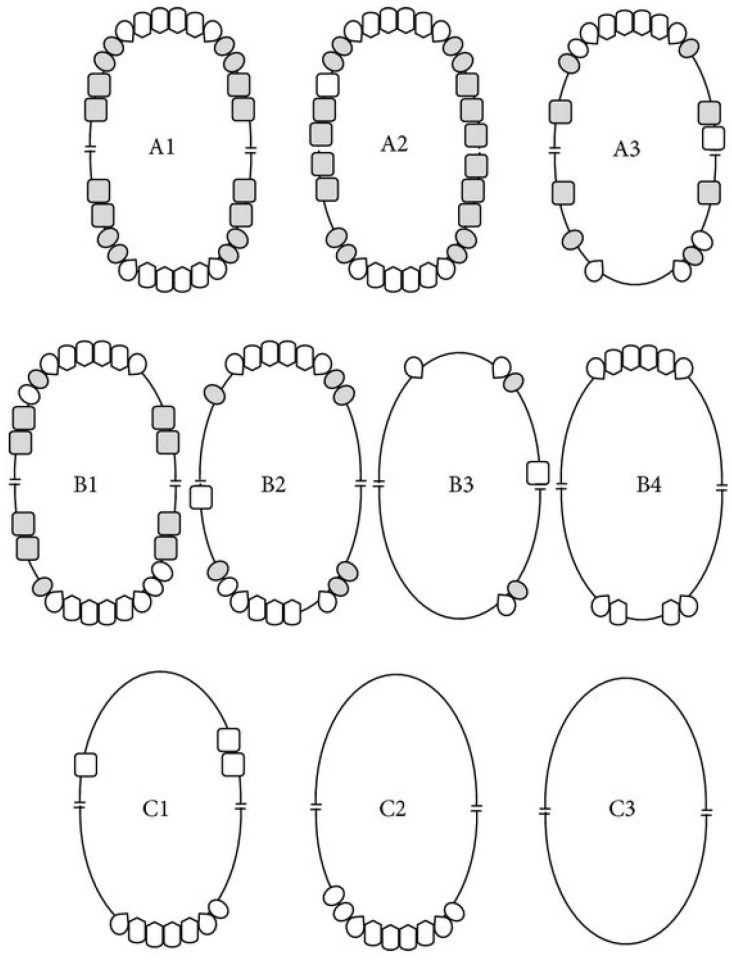
This is a schematic representation of Eichner’s classification [22]. The shaded teeth indicate the occlusal contacts between the natural teeth or fixed prostheses in the premolar and molar regions, which constitute the occlusal support zones (OSZs). Category A contains four OSZs. A1: Complete dentition. A2: Missing teeth in one arch. A3: Missing teeth in both arches. Category B contains one to three OSZs or contacts in the anterior area only. B1: Three OSZs. B2: Two OSZs. B3: One OSZ. B4: Contacts in the anterior area only. Category C contains no OSZs. C1: Teeth in both arches. C2: Teeth in one arch only. C3: Edentulous [22].

During their next visit to the memory clinic, an oral and radiographic (orthopantomogram, bitewings) screening was performed by dentists. To ensure consistency, all assessors followed the same assessment procedures. The following oral data was collected:

- Caries lesions (number and severity) were scored using the International Caries Detection and Assessment System (ICDAS). ICDAS is a standardized visual scoring system that classifies the severity of tooth decay from initial enamel changes (scores 1–3) to established cavitated lesions (scores 4–6) [19]. In the present study, scores of 4–6 were considered to represent clinically relevant caries, as these lesions involve the dentine and generally require restorative treatment. Early enamel lesions (scores of 1–3) were excluded in order to focus on manifest caries with direct clinical implications and to enable a reliable assessment to be made within the limited time available for examination in the memory clinic setting.
- Periodontal pockets (number of pockets with a depth of ≤3 mm, 4–5 mm and ≥6 mm) [20].
- (Suspicion) of periapical lesions (as far as they could be detected on the radiographs).
- Number of occlusal units according to the Eichner Index (this index classifies occlusal support based on the number and location of contacts in the premolar and molar regions) (Figure 1). A minimum of four occlusal units is required to achieve adequate masticatory function [21].
- Chewing ability was assessed using a single self-reported item. Responses were recorded on a five-point ordinal scale ranging from ‘very good’ to ‘poor’, with an additional ‘do not know’ option (See Appendix A.1).
- Eichner’s classification (patients are classified into one of ten groups (A1, A2, A3, B1, B2, B3, B4, C1, C2 and C3) (based on the Eichner’s index) (see Figure 1)).
- Number of broken teeth.
- Xerostomia (yes/no).
- Oral self-care (frequency of brushing and tools to clean oral cavity and protheses edentulousness (yes/no).
- Gingivitis (swollen and red gums).
- Mucosal pathology (canker sores, candidiasis, linea alba).
- Bone pathology [22].

### 2.5. Sample Size Calculation

The required sample size was determined using G*Power version 3.1.9.4. The power analysis was performed with an alpha level of 0.025 (0.05/2), 80% power (β = 0.20) and an expected effect size of 0.31 [23,24]. Based on these parameters, the minimum required sample size was 110 patients.

### 2.6. Statistical Analyses

The Shapiro–Wilk test was used to determine the distribution of the data. For all variables that were not normally distributed, the median scores and interquartile ranges (IQR) were reported. For data with a normal distribution, mean value and standard deviations (SD) were reported.

Descriptive statistics were used to report demographics, health and oral health. Chi-square tests, Fisher’s Exact Test and Mann–Whitney U tests were used to analyze differences between subgroups of cognitive complaints and oral status. A *p*-value of <0.05 was considered statistically significant.

All statistical analyses were performed using SPSS IBM Statistics version 23.0 (SPSS, Chicago, IL, USA) and R-studio (2025.05.1 + 513).

## 3. Results

### 3.1. Participants

Supplementary Table S1 gives an overview of all patients who were seen in the memory clinic and were included and those who could not or did not want to participate. Between April 2022 and May 2025, 230 consecutive new patients who had an appointment for their first consult at the memory clinic were asked to participate in this study. A total of 144 patients (62.6%) enrolled in this study; 73 (31.7%) were not willing to participate for various reasons such as no interest, too anxious about their memory complaints, no energy to participate, and not willing to participate. A further five (2.1%) patients were bedridden and too ill to participate, four (1.7%) patients did not want to undergo dental radiographs because they feared radiation, and four (1.7%) patients were incapacitated to consent. On average, non-participating patients were significantly older than participating patients (mean age: 76.2 vs. 73.7 years; *p* = 0.003). Smoking and alcohol use were reported more frequently in the non-participant group than in the participating (*p* = 0.003 and *p* = 0.004, respectively). Furthermore, non-participants had lower MMSE scores, indicating greater cognitive impairment (*p* = 0.026).

### 3.2. Dementia

Table 2 shows the differences between patients who were diagnosed with dementia (*n* = 63; 44%) and those who were not diagnosed with dementia (*n* = 81; 56%). On average, participants with dementia were slightly older (74.9 years, SD 5.9) than those without (72.7 years, SD 7.7). Overall, the demographic and comorbidity profiles of the two groups appeared comparable with the exception of the MMSE score (*p* = 0.0004).

### 3.3. Oral Status and Oral Health Related Quality of Life (OHIP-14)

A variation in oral status characteristics was observed within the group of participants, but no significant differences could be observed between participants with or without dementia (Table 3). Three-quarters of the participants (75.0%) had natural teeth, with an average of 15.8 natural teeth (SD 11.0). One in four participants (*n* = 36, 25.0%) was edentulous. Complete maxillary dentures were present in 32.6% of participants (part of the patients were edentulous in the maxilla but had natural teeth in the mandible), while complete mandibular dentures were present in 25.0%. Partial removable dentures and implant-retained overdentures were reported less frequently.

The mean OHIP-14 score across the total cohort was 18.4 (SD 6.0). In line with oral status, no statistically significant differences were observed with respect to the OHIP scores between participants with and without dementia.

### 3.4. Occlusion, Occlusal Support (Eichner Index) and Self-Reported Chewing Problems

**Figure 2:**
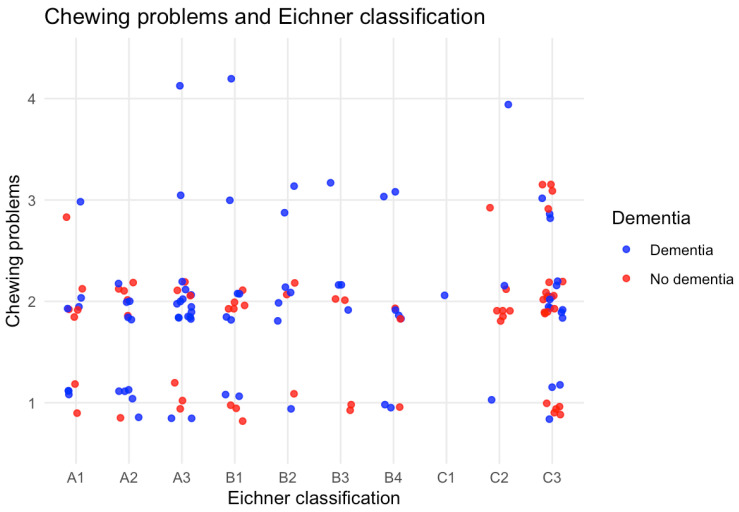
Chewing problems and Eichner Classification. The relationship between self-reported chewing problems and the Eichner index. The x-axis shows the Eichner classification (A–C), which indicates the level of occlusal support according to the number of posterior occlusal contacts. The y-axis shows per dot the number of patients who reported masticatory problems (1 = Very good, 2 = Good, 3 = Moderate and 4 = Bad). Higher Eichner classification (A) reflects sufficient occlusal support, whereas lower classes (B,C) indicate reduced or absent support. The Figure visually suggests that the prevalence of masticatory problems increases as occlusal support decreases. However, no statistically significant correlation was found.

Table 4 shows the occlusal units in participants with a natural dentition. Over half of the participants were very limited in the occlusal support as 52.8% had only 0 to 3 occlusal units. The less occlusal support, the more chewing problems were reported (Figure 2). No differences in occlusal support were seen between participants diagnosed with or without dementia (Table 4 and Table 5). Based on the Eichner classification in Table 5, less than half of the group (45.1%) fell into categories indicating partial or no posterior occlusal support (B3, B4, C1, C2, C3), making the ability to chew more difficult. While category C3 (no occlusal contact in any of the four support zones) was the most prevalent in both groups, there were no statistically significant differences between participants with or without dementia (Table 5). Also, despite apparent visual differences, no statistically significant associations were found between self-reported chewing problems and the Eichner classification between both groups (Figure 2).

### 3.5. Oral Self-Care of the Participants

Table 6 shows participants’ self-reported daily oral hygiene routines. Among participants with natural dentition (*n* = 108), the majority reported brushing their teeth twice daily or more (72.2%), with no significant difference between those with (72.7%) and without (71.9%) dementia. Manual toothbrush use was more prevalent overall (62.0%) than electric toothbrush use (44.4%). Participants diagnosed with dementia were more likely to use a manual toothbrush (72.2% vs. 54.7%), whereas those without dementia were more likely to use an electric toothbrush (48.4% vs. 38.6%). Some participants use both manual and electric toothbrushes. However, these differences were not statistically significant. Although all participants were active in oral cleaning, plaque scores were rather high (see oral health problems further on in this article). Interdental cleaning was reported by 45.4% of dentate participants, while 23.1% reported using mouthwash, with no significant differences between groups.

Among the edentulous participants (*n* = 36), the majority brushed once daily (66.0%), whereas brushing twice daily was less common (34.0%). Electric toothbrush use was rare in this group (5.6%), with a strong preference for manual brushing (97.2%). Mouthwash use was low (11.1%) and did not differ between groups. No significant differences in oral hygiene behaviour were observed between edentulous participants with and without dementia.

### 3.6. Oral Health Problems

Table 7 shows the oral health problems among the participants with and or without a natural dentition. Oral health problems were common in the total study population of 144 participants. Among participants with natural dentition (*n* = 108), at least one oral health problem was recorded. Carious teeth were present in 93.5% of this group, with an average of 3.62 (SD 2.6) carious teeth per person. Deep carious lesions (ICDAS score ≥ 4) were observed in 64.8% of participants with a natural dentition. Periodontal pockets measuring 4–5 mm were found in 20.4% of dentate participants and pockets measuring ≥6 mm in 76.9%. Residual roots and broken teeth were present in 11.1% and 12.0% of participants, respectively. The mean plaque score was 1.36 (SD 0.93), and gingivitis was present in 32.4% of cases although all participants claimed to brush once or twice a day. It is possible that some periodontal pockets are actually long-standing chronic lesions with reduced inflammatory activity. These pockets may result in limited bleeding despite substantial attachment loss. No statistically significant differences were found when comparing participants with and without dementia within the dentate group.

Among the participants with dentures (*n* = 47), denture-related problems were highly prevalent (61.7%). The distribution of problems with maxillary and mandibular dentures was comparable between groups. Combined problems with both upper and lower dentures were reported by 26.9% of participants with dementia and 19.0% of those without.

Self-reported chewing problems were present in 13.9% of participants and xerostomia in 14.6%. These outcomes did not differ significantly between groups.

## 4. Discussion

This study examined the oral health of older adults with memory impairments who were attending a memory clinic for a CGA. The main finding was that oral health problems were highly prevalent in this group, regardless of whether participants were diagnosed with dementia or not. Deep carious lesions (93.5%) and periodontal problems (97.3%) were particularly prevalent among individuals with teeth, while those without teeth frequently experienced denture-related issues (61.7%). These results suggest that poor oral health is present in older adults with memory complaints, with no significant differences between older people with memory complaints and those who are diagnosed with dementia.

The prevalence of oral disease found in this study is higher than that reported in the general older adult population [25,26]. Previous population-based studies [1,4,5,6] have shown that approximately 30–40% of older adults have untreated caries or periodontal disease; however, in this study, over 60% of participants had deep carious lesions, and almost 80% had periodontal pockets. This suggests that individuals referred to a memory clinic indeed may be particularly vulnerable, possibly due to reduced (oral) self-care, multimorbidity, and medication use affecting oral health [12,13]. Cognitive decline can make oral hygiene more challenging, but even in the pre-dementia phase, executive dysfunction, reduced motivation, and less-structured routines may already contribute to the neglect of oral (self) care [27]. Furthermore, the results highlight the importance of incorporating oral health assessment into routine assessments at memory clinics or geriatric care centres, given the high prevalence of untreated tooth decay, periodontal issues, denture problems, and reduced occlusal support. These problems will worsen rapidly if they remain undetected and cause a threat for overall health and (social) wellbeing. Specifically, participants with natural dentitions had oral health problems more often which could interfere with general health, as deep carious lesions and periodontal problems pose a high risk of infection [27,28] and pain or chewing problems to the participants.

The comparable oral health status of participants with and without dementia is consistent with earlier studies indicating that deterioration in oral health begins at an early stage of cognitive decline. The literature showed that mild cognitive impairment and subjective memory complaints are associated with poorer oral health and worse oral hygiene behaviour [28]. This may explain why no significant difference was observed between the groups in this study. It also highlights that oral health problems in this population should not only be regarded as a consequence of cognitive decline but as a relevant issue throughout the cognitive decline continuum [27]. It should also be noted that many of these individuals are likely to receive support with their daily hygiene, which may mask differences that would otherwise become more apparent without assistance.

In terms of subjective oral health, the mean OHIP-14 score indicated a moderate impact on oral health-related quality of life, which is consistent with previous studies in older populations with multimorbidity [18]. Older people often consider oral health issues not as a real problem as they often have many more problems that are far more serious such as mobility problems and cognitive problems. Oral health problems do not always provoke pain, e.g., periodontal problems stay there usually undetected. Furthermore, the use of painkillers is not uncommon in older people, thereby interfering with the body’s notification system in case of health threats.

The observed differences in oral self-care behaviours and the high plaque scores suggest early signs of deterioration of self-reliance in oral hygiene practices. Manual toothbrushing was significantly more common among participants with dementia, whereas electric toothbrushes were more commonly used by those who were not (yet) diagnosed with dementia. This may indicate a regression to simpler, more familiar routines, or a lack of support in maintaining more effective oral hygiene methods [29,30].

From a health policy perspective, these findings support the integration of oral health assessments into the CGA, particularly in memory clinics. While the current CGA covers physical, cognitive, social and functional domains, it omits oral health, despite its well-documented links to systemic health, nutrition, infection risk and quality of life [5,6,13]. In order to incorporate oral health into a CGA, a multidisciplinary approach is required involving dentists, geriatricians and nurses. Regular oral screenings should be incorporated into routine evaluations, with clear referral pathways to dental professionals in the event of any issues being identified [31]. Simply noting inadequate oral hygiene or care is often not acted upon, meaning more than reporting alone is needed [31]. Healthcare providers should be trained to recognize oral health issues and support daily oral hygiene, for example, by providing adapted oral care tools. Involving and educating caregivers is also crucial, as they play a key role in maintaining oral care. Finally, developing simple communication protocols between dental and medical teams would help to ensure continuity of care and early intervention, ultimately improving the overall health and quality of life of this vulnerable population.

### Strengths and Limitations

A strength of this study is that this is the first study, as far as we know, that assesses oral health of older people with memory complaints visiting a memory clinic. As the results show that dental health problems are frequently seen among this population, it provides a data-driven rationale that including oral health assessments into a CGA could help detect oral health issues that would remain undetected in this vulnerable population, as these oral health issues can cause severe health problems in the near future.

However, there are also some limitations. A large number of eligible participants were either unable or unwilling to take part. As this group generally has more health problems and lower MMSE scores, it is likely that their oral health is no better than that of the participants who were included. Therefore, our findings may underestimate the true situation. Another limitation to notice is the reliance on self- or proxy-reported questionnaire outcomes. Given the presence of memory complaints, it is difficult to ascertain whether the patient actually carried out the self-reported oral hygiene behaviours. Proxy responses may not accurately reflect patients’ actual daily routines or oral health practices. Connection with home care staff as additional informants could improve the accuracy of proxy-reported data, particularly in cases where there is no consistent informal carer. This study was conducted in a memory clinic, which limits how widely the findings can be generalized to the wider older population. Consequently, Berkson’s bias may be present, as clinic attendees are likely to differ systematically from the general population in terms of health status and care needs. Furthermore, due to the observational and cross-sectional nature of the study, it is not possible to establish causal relationships. For example, it is impossible to determine whether memory problems contribute to poor oral health, or if underlying factors influence both conditions. Further longitudinal studies are needed to explore the temporal relationships and potential causal pathways between cognitive decline and oral health. Lastly, important lifestyle and behavioural factors, such as alcohol consumption, smoking, and powered toothbrush use, were not considered when categorizing patients. This was done with a reason to avoid false outcomes. Alcohol and tobacco are known to influence oral health and patterns of oral disease; however, if oral self-care is good, this effect is small. Electric toothbrush use is counted if the electric brush is used properly, and that is often hard for this population.

## 5. Conclusions

Oral health issues are high prevalent among older people with memory complaints visiting the memory clinic, with no significant differences between patients diagnosed with or without dementia. Therefore, integrating structured oral health assessments into the CGA would offer opportunities for early detection and intervention to prevent further decline or oral health which, in turn, could prevent general health problems and social wellbeing. Dentate patients mainly experience other problems than edentulous patients. Tooth-related problems such as broken teeth, infection, pain, and periodontal problems are not seen in edentulous patients who have other problems such as loose dentures or ulcers in the mucosa. Oral healthcare therefore should be tailored according to oral status (dentulous versus edentulous) in order to optimize prevention and treatment.

## Figures and Tables

**Table 1:** Overview of the included test performed during the conventional CGA (comprehensive geriatric assessment) at the memory clinic of the UMCG including a somatic, mental, social and functional assessment.

	Somatic	Mental	Functional	Social
Anamnesis	- Anamnesis - Medical history - Medication usage (polypharmacy) - Mini Nutritional Assessment [15]	- Anamnesis - Assessment of psychological functioning (cognition, behaviour, attention and mood)	- Katz ADL [16] - Continence	- Home situation (marital status, children and occupation - Social activities
Physical examination	- Tract anamnesis (general, circulatory, respiratory, digestive, genitourinary, musculo-skeletal, cerebrospinal, endocrine and psychiatric) - Full neurological assessment (mood, speech and language, orientation, perceptual disturbances, motor skills, coordination, sensitivity, reflexes and the cranial nerves		- Sensory Functions (evaluate vision, hearing, and other senses like taste and smell) - Mobility (balance, condition and strength)	
Additional examination	- Diagnostic imaging - Blood test	- Neuropsychological assessment (MMSE [16]/MOCA [17]) - Psychiatric examination		- Additional hetero anamnesis

**Table 2:** Characteristics of the participants of the different groups.

Participants Characteristics	Total (*n* = 144)	Participants with Dementia (*n* = 63)	Participants with Memory Complaints but Not Diagnosed with Dementia (*n* = 81)	*p*-Value
Gender (*n*, %)				
Female	58 (40.3)	29 (46.0)	29 (35.8)	0.284 *
Male	86 (59.7)	34 (54.0)	52 (64.2)	0.284 *
Age, year (mean, SD)	73.7 (7.0)	74.9 (5.9)	72.7 (7.7)	0.051 ’
Polypharmacy (*n*, %)	74 (51.4)	30 (47.6)	44 (54.3)	0.529 *
Smoking (*n*, %)	77 (53.5)	34 (54.0)	43 (53.1)	1.000 *
Alcohol use (*n*, %)	104 (72.1)	50 (79.4)	54 (66.7)	0.134 *
KATZ ADL, median (IQR)	2.0 (4.0)	3.0 (4.0)	1.0 (4.0)	0.058 ^
Living at home (*n*, %)	141 (97.9)	61 (96.8)	80 (98.8)	0.581 ^#^
Most mentioned comorbidities				
- Hypertension (*n*, %)	53 (36.8)	22 (34.9)	31 (38.3)	0.811 *
- Diabetes mellitus (*n*, %)	28 (19.4)	9 (4.8)	19 (23.5)	0.243 *
- Depression (*n*, %)	20 (13.9)	6 (9.5)	14 (17.3)	0.274 *
- Cerebral ischemic attack (*n*, %)	16 (11.1)	9 (14.3)	7 (8.6)	0.423 *
- Atrial fibrillation (*n*, %)	14 (9.7)	3 (4.8)	11 (13.6)	0.300 ^#^
- Chronic obstructive (*n*, %) pulmonary disease (COPD) (*n*, %)	13 (9.0)	5 (7.9)	8 (9.9)	0.913 *
- High cholesterol (*n*, %)	13 (9.0)	5 (7.9)	8 (9.9)	0.913 *
MMSE, median (IQR)	26.0 (7.0)	24.0 (7.0)	27.0 (6)	**0.0004 ^**

* Chi-square test, ^ Wilcoxon Rank Sum test, ’ independent samples *t*-test, ^#^ Fisher’s exact test, the bold means the data is significant.

**Table 3:** Oral status of the participants diagnosed with and without dementia.

Patient Characteristics	Total Number of Participants (*n* = 144)	Participants with Dementia (*n* = 63)	Participants with Memory Complaints Not Diagnosed with Dementia (*n* = 81)	*p*-Value
Natural dentition, (*n*, %)	108 (75.0)	44 (69.8)	64 (79.0)	0.286 *
Number of teeth, median, (IQR)	19.5 (23.5)	17.0 (24.0)	21.0 (15.0)	0.064 ^
A maxillary partial denture (*n*, %)	12 (8.3)	6 (9.5)	6 (7.4)	0.879 *
A mandibular partial denture (*n*, %)	12 (8.3)	7 (11.1)	5 (6.2)	0.447 *
Edentulous elderly (*n*, %)	36 (25.0)	19 (30.2)	17 (21.0)	0.286 *
A complete maxillary denture (*n*, %)	47 (32.6)	26 (41.3)	21 (25.9)	0.077 *
A complete mandibular denture (*n*, %)	36 (25.0)	19 (30.2)	17 (21.0)	0.286 *
Implant-retained overdenture (*n*, %)	17 (11.8)	8 (12.7)	9 (11.1)	0.974 *
OHIP-14 (median, IQR)	16.0 (6.3)	16.0 (6.5)	15.0 (6.0)	0.632 ^

* Chi-square test, ^ Wilcoxon Rank Sum test.

**Table 4:** Occlusal units in participants with natural dentition.

Occlusal Units	Total Number of Participants (*n* = 108)	Participants with Dementia (*n* = 44)	Participants with Memory Complaints Not Diagnosed with Dementia (*n* = 64)	*p* Value
0–3 (*n*, %)	40 (37.0)	17 (38.6)	23 (35.9)	0.934 *
4–12 (*n*, %)	68 (63.0)	27 (61.4)	41 (64.1)	0.934 *

* Chi-square test.

**Table 5:** Distribution of Eichner classification among subgroups.

Eichner Index	Total Number of Participants (*n* = 144)	Participants with Dementia (*n* = 63)	Participants with Memory Complaints Not Diagnosed with Dementia (*n* = 81)	*p* Value
A1 (*n*, %)	14 (9.2)	7 (11.1)	7 (8.6)	0.832 *
A2 (*n*, %)	16 (11.1)	6 (9.5)	10 (12.3)	0.789 *
A3 (*n*, %)	23 (16.0)	6 (9.5)	17 (21.0)	0.102 *
B1 (*n*, %)	16 (11.1)	8 (12.7)	8 (9.9)	0.790 *
B2 (*n*, %)	10 (6.9)	3 (4.8)	7 (8.6)	0.514 ^#^
B3 (*n*, %)	8 (5.6)	4 (6.3)	4 (4.9)	0.730 ^#^
B4 (*n*, %)	10 (6.9)	3 (4.8)	7 (8.6)	0.514 ^#^
C1 (*n*, %)	1 (0.7)	0 (0.0)	1 (1.2)	1.000 ^#^
C2 (*n*, %)	10 (6.9)	7 (11.1)	3 (3.7)	0.104 ^#^
C3 (*n*, %)	36 (25.0)	19 (30.2)	17 (21.0)	0.286 *

* Chi-square test, ^#^ Fisher’s exact test.

**Table 6:** Oral self-care of the participants.

Participants with Natural Dentition				
	Total Number of Participants (*n* = 108)	Participants with Dementia (*n* = 44)	Participants with Memory Complaints Not Diagnosed with Dementia (*n* = 64)	*p*-Value
Frequency of tooth brushing per day (*n*, %)				
- ≥2	78 (72.2)	32 (72.7)	46 (71.9)	1.000 *
- 1	30 (27.8)	12 (27.3)	18 (28.1)	1.000 *
- Not brushing	0 (0.0)	0 (0.0)	0 (0.0)	
Toothbrush (*n*, %)				
- Electric toothbrush	48 (44.4)	17 (38.6)	31 (48.4)	0.418 *
- Manual toothbrush	67 (62.0)	32 (72.7)	35 (54.7)	0.090 *
Assisted with tooth brushing.	6 (5.5)	2 (4.5)	4 (6.3)	1.000 ^#^
Interdental cleaning (*n*, %)	49 (45.4)	18 (40.9)	31 (48.4)	0.565 *
Mouthwash, (*n*, %)	25 (23.1)	13 (29.5)	12 (18.8)	0.191 *
**Participants with Dentures**	**Total Number of Participants (*n* = 47)**	**Participants with Dementia (*n* = 26)**	**Participants with Memory Complaints Not Diagnosed with Dementia (*n* = 21)**	***p*-Value**
Frequency of tooth brushing per day (*n*, %)				
- ≥2	16 (34.0)	7 (26.9)	9 (42.9)	0.403 *
- 1	31 (66.0)	19 (73.1)	12 (57.1)	0.403 *
- Not brushing	0 (0.0)	0 (0.0)	0 (0.0)	
Toothbrush (*n*, %)				
- Electric toothbrush	6 (5.6)	5 (19.2)	1 (4.8)	0.204 ^#^
- Manual toothbrush	43 (97.2)	26 (100.0)	17 (81.0)	**0.034 ^#^**
Mouthwash (*n*, %)	6 (11.1)	3 (11.5)	3 (14.3)	1.000 ^#^

* Chi-square test, ^#^ Fisher’s exact test, the bold means the data is significant.

**Table 7:** Oral health problems of the participants.

Participants with Natural Dentition				
	Total Number of Participants with a Natural Dentition (*n* = 108), *n* (%)	Participants with Dementia (*n* = 44), *n* (%)	Participants with Memory Complaints Not Diagnosed with Dementia (*n* = 64), *n* (%)	*p*-Value
Carious teeth	101 (93.5)	40 (90.9)	61 (95.3)	0.440 ^#^
Number of carious teeth per participant, mean (SD)	3.0 (3.0)	3.5 (2.5)	3.0 (3.0)	0.317 ^
Deep carious lesions (ICDAS 4 and up)	70 (64.8)	29 (65.9)	41 (64.1)	1.000 *
Periodontal pockets 4–5 mm	23 (21.3)	13 (29.5)	10 (15.6)	0.134 *
Periodontal pockets ≥ 6 mm	85 (78.7)	31 (70.5)	54 (84.4)	0.134 *
Residual roots	12 (11.1)	5 (11.4)	7 (10.9)	1.000 *
Broken teeth	13 (12.0)	5 (11.4)	8 (12.5)	1.000 *
Plaque score, median (IQR)	1.2 (1.7)	1.2 (1.8)	1.1 (1.6)	1.000 ^
Gingivitis (swollen and red gums started bleeding on probing))	36 (33.3)	18 (40.9)	18 (28.1)	0.239 *
**Participants with Dentures**	**Total Number of Participants (*n* = 47), *n* (%)**	**Participants with Dementia (*n* = 26), *n* (%)**	**Participants with Memory Complaints not Diagnosed with Dementia (*n* = 21), *n* (%)**	***p*-Value**
Complete and partial denture problems:	29 (61.7)	16 (61.5)	13 (61.9)	1.000 *
Maxillary denture	13 (27.7)	5 (19.2)	8 (38.1)	0.733 *
Mandibular denture	5 (10.6)	4 (15.4)	1 (4.8)	0.338 ^#^
Both dentures	11 (23.4)	7 (26.9)	4 (19.0)	1.000 ^#^
**All Participants**	**Total Number of Participants (*n* = 144), *n* (%)**	**Participants with Dementia (*n* = 63), *n* (%)**	**Participants with Memory Complaints Not Diagnosed with Dementia (*n* = 81), *n* (%)**	***p*-Value**
Mucosal pathology	24 (16.7)	8 (12.7)	16 (19.8)	0.367 *
Bone pathology:				
Periapical radiolucencies	52 (36.1)	22 (36.7)	30 (35.9)	0.930 *
Periapical opaque lucencies	2 (1.4)	2 (3.2)	0 (0)	0.190 ^#^
Chewing problems	20 (13.9)	6 (9.5)	14 (17.3)	0.274 *
Xerostomia	21 (14.6)	6 (9.5)	15 (18.5)	0.201 *

* Chi-square test, ^ Wilcoxon Rank Sum test, ^#^ Fisher’s exact test.

## Data Availability

All data generated or analyzed during this study are included in this article. Further enquiries can be directed to the corresponding author.

## Supplementary Materials

The following supporting information can be downloaded at: https://www.mdpi.com/article/10.3390/ijerph23020212/s1, Table S1. Characteristics of the participants (participating versus not participating patients).

## Appendix A

### Appendix A.1. Questionnaire Oral Situation

1. Do you regularly experience inflammation of your teeth, oral mucosa, and/or gums?
  (This may be noticeable as pain, redness, or easy bleeding of the gums)

□ Yes, I experience inflammation every day

□ Yes, I occasionally experience inflammation of the oral mucosa or gums

□ No, I rarely or never experience inflammation in my mouth

□ I do not know

2. Do you suffer from a dry mouth?

During the day:

□ No, never

□ No, rarely

□ Yes, sometimes

□ Yes, often

□ I do not know

At night:

□ No, never

□ No, rarely

□ Yes, sometimes

□ Yes, often

□ I do not know

3. Do you usually need to drink liquids when eating dry foods?

□ Yes

□ No

□ I do not know

4. How often do you brush your teeth or clean your dentures?

□ Three times a day or more

□ Twice a day

□ Once a day

□ Occasionally, but not every day

□ Never

□ I do not know

5. Are you able to do this yourself, or do you receive assistance?

□ I do this myself

□ Someone else assists me

□ I do not know

6. What do you use to brush your teeth?

□ Electric toothbrush

□ Manual toothbrush

□ I do not brush my teeth

7. Do you use any aids to clean between your teeth?

□ Yes

□ No → proceed to question 9

8. What do you use to clean between your teeth?

□ Interdental brushes

□ Toothpicks

□ Dental floss

□ Other, namely: …………………

9. Do you experience unpleasant or bad breath?

□ Never

□ Sometimes

□ Regularly

□ Often

□ Almost always

□ I do not know

10. How severe is the unpleasant or bad breath you experience?

□ Not at all

□ Mild

□ Moderate

□ Severe

□ Very severe

□ I do not know

11. Do you use a mouth rinse or mouthwash?

□ Yes, namely: …………………………………

□ No

12. When was your most recent dental check-up with a dentist, dental hygienist, or other dental care professional?

□ Less than 1 year ago

□ 1–2 years ago

□ More than 2 years ago

□ I never visit a dental care professional

□ I do not know

13. How long ago did you most recently experience pain in your mouth
  (e.g., jaw, tongue, teeth, molars, implants, oral mucosa, etc.)?

□ Recently (less than 3 months ago)

□ More than 3 months ago

□ I have never experienced oral pain → proceed to question 14

□ I do not remember → proceed to question 14

14. How did the pain complaint progress?

□ I no longer have pain; I did nothing and the pain resolved spontaneously

□ I no longer have pain; I took pain medication and the pain resolved

□ I no longer have pain; I visited a dentist or general practitioner and the pain resolved

□ I still have (regular) pain; I am currently doing nothing about it

□ I still have (regular) pain; I take pain medication

□ I still have (regular) pain; I visited a dentist or general practitioner

□ I still have (regular) pain; I plan to make an appointment with a dentist or general practitioner

□ I do not remember

15. Are you able to chew properly?

□ Very well

□ Well

□ Moderately

□ Poorly

□ I do not know

16. How would you rate the current condition of your teeth and/or dentures?

□ Excellent

□ Good

□ Moderate

□ Poor

□ I do not know

17. Do you avoid social contacts because of problems with your teeth and/or dentures?

□ Never

□ Occasionally

□ Regularly

□ Often

□ I do not know

18. On a scale from 1 to 10, how would you rate your own teeth and/or dentures?
  (Please circle one number)

1 2 3 4 5 6 7 8 9 10

19. When visiting the dentist, are you able to attend independently or do you require assistance?

□ Yes, completely independently (e.g., driving a car, motorcycle, moped, cycling, or walking)

□ Yes, independently using public transportation

□ No, others such as family members, partners, or friends accompany or transport me because I am no longer able to attend independently

□ I do not know

20. Who is your dentist (so that we can send the radiographs to them)?

…………………………………………………………………………………………………

### Appendix A.2. Oral Health Impact Profile (OHIP-14)

1. Have you had trouble pronouncing any words because of problems with limitation your teeth, mouth or dentures?

* Never

* Hardly ever

* Occasionally

* Fairly often

* Very often

2. Have you felt that your sense of taste has worsened because of problems with your teeth, mouth or dentures?

* Never

* Hardly ever

* Occasionally

* Fairly often

* Very often

3. Have you had painful aching in your mouth?

* Never

* Hardly ever

* Occasionally

* Fairly often

* Very often

4. Have you found it uncomfortable to eat any foods because of problems with your teeth, mouth or dentures?

* Never

* Hardly ever

* Occasionally

* Fairly often

* Very often

5. Have you been self-conscious because of your teeth, mouth or dentures?

* Never

* Hardly ever

* Occasionally

* Fairly often

* Very often

6. Have you felt tense because of problems with your teeth, mouth or dentures?

* Never

* Hardly ever

* Occasionally

* Fairly often

* Very often

7. Has your diet been unsatisfactory because of problems with your teeth, disability with your mouth or dentures?

* Never

* Hardly ever

* Occasionally

* Fairly often

* Very often

8. Have you had to interrupt meals because of problems with your teeth, mouth or dentures?

* Never

* Hardly ever

* Occasionally

* Fairly often

* Very often

9. Have you found it difficult to relax because of problems with your teeth, disability with your mouth or dentures?

* Never

* Hardly ever

* Occasionally

* Fairly often

* Very often

10. Have you been a bit embarrassed because of problems with your teeth, mouth or dentures?

* Never

* Hardly ever

* Occasionally

* Fairly often

* Very often

11. Have you been a bit irritable with other people because of problems with disability of your teeth, mouth or dentures?

* Never

* Hardly ever

* Occasionally

* Fairly often

* Very often

12. Have you had difficulty doing your usual jobs because of problems with your teeth, mouth or dentures?

* Never

* Hardly ever

* Occasionally

* Fairly often

* Very often

13. Have you felt that life in general was less satisfying because of problems with your teeth, mouth or dentures?

* Never

* Hardly ever

* Occasionally

* Fairly often

* Very often

14. Have you been totally unable to function because of problems with your teeth, mouth or dentures?

* Never

* Hardly ever

* Occasionally

* Fairly often

* Very often
