# Supplementary material for: Value of Oral Health Assessments for Older People with Memory Complaints Visiting a Memory Clinic for a Comprehensive Geriatric Assessment: A Cross-Sectional Study
Source: Int J Environ Res Public Health. 2026 Feb 9;23(2):212. doi: 10.3390/ijerph23020212 (PMC12941030; doi:10.3390/ijerph23020212)
Supplement: Supplementary file 1 [file ijerph-23-00212-s001.zip › ijerph-4085492-supplementary.pdf]

Supplementary Materials:

Table S1. Characteristics of the participants (participating versus not participating patients)

**Patient characteristics, N=230**	**Participating, N = 144**	**Not participating, N = 86**	**P-value**
Gender; male (n %)	86 (59.7)	47 (54.7)	0.538*
Age, year (median, IQR)	73.0 (8.25)	77.0 (6.8)	***0.003^***
Polypharmacy (n, %)	74 (51.4)	55 (64.0)	0.085*
Smoking (n, %)	77 (53.5)	28 (32.6)	***0.003****
Alcohol use (n, %)	104 (72.1)	45 (52.3)	***0.004****
**Top 5 comorbidities:**			
- Diabetes mellitus (n, %)	28 (19.4)	27 (31.4)	0.058*
- Chronic obstructive pulmonary disease (COPD) (n, %)	13 (9.0)	13 (15.1)	0.232*
- Hypertension (n, %)	53 (36.8)	40 (46.5)	0.189*
- High cholesterol (n, %)	13 (9.0)	16 (18.6)	0.056*
MMSE, median (IQR)	26.0 (7.0)	24.0 (6.8)	***0.026^***
